# Supplementary material for: Patients’ Anticipation for the Pharmacies of Rural Communities: A Qualitative Study from Pakistan
Source: Int J Environ Res Public Health. 2019 Jan 7;16(1):143. doi: 10.3390/ijerph16010143 (PMC6338954; doi:10.3390/ijerph16010143)
Supplement: Supplementary file 1 [file ijerph-16-00143-s001.zip › ijerph-404143 - supp 1.pdf]

Interview guide

Demographics

Age:______________________________ Gender: ______________________________

Education status: ____________________ Monthly income: _______________________

Reason of Visit/Disease: ___________________________

A. Current Services

1. How you find the rural pharmacies as compare to urban pharmacies ?
2. What is your opinion about premises and area of pharmacies in your locality ?
3. How the working of pharmacies in your locality justifies the need of patients?
4. Does the pharmacy maintains high standard of desired medicines ?
5. How do you ranks the education and training of staff to their current job?
6. How the pharmacy manager/ owner / staff behaves to patients ?
7. What do you think about the staffing of pharmacy in your locality?
8. How the medicines are dispensed ?
9. How the staff of pharmacies inform you about the use of medicines ?
10. What are the major problems in the current services of pharmacy ?
11. What additional facilities are provided by pharmacies

B. Suggestions

12. Do you expect any type of more services ?
13. How can be services improved?
14. Which stake holder like drug inspector, doctor, pharmacy owner can majorly contribute to improve services?
