# Supplementary material for: Patients’ Anticipation for the Pharmacies of Rural Communities: A Qualitative Study from Pakistan
Source: Int J Environ Res Public Health. 2019 Jan 7;16(1):143. doi: 10.3390/ijerph16010143 (PMC6338954; doi:10.3390/ijerph16010143)
Supplement: Supplementary file 1 [file ijerph-16-00143-s001.zip › ijerph-404143 - supp 3.pdf]

Permission and Information Sheet/ Pharmacies

Patients' Anticipation for the Pharmacies of Rural Communities: A Qualitative Study from Pakistan

Interviewer: ______________________

Purpose of the study: The purpose of this study is to grasp the knowledge about the "Pharmacies of Rural Communities". We will analyze that how the people perceive the services of pharmacies in rural communities. It means to have in-depth understandings of function and structure with respect to patients need .

Methodology: A qualitative study will perform to gather information about rural pharmacies. We will conduct semi-structured interview to know societal perception and real need of the patients.

Confidentiality:

The interview will be conducted face to face. The information gathered from your customer/s will remain confidential and only the researchers will have access to it. The name of pharmacy or owner will not be used anywhere in the study.

Before agreeing to this study, it is important that you have clearly understood the purpose of the study. This agreement states that you have understood everything about the study and that you are giving us permission to use information gathered from your customer/s .

Date: _____________
